# Evidence and implication of interventions across various socioecological levels to address pre-exposure prophylaxis uptake and adherence among men who have sex with men in the United States: a systematic review

**DOI:** 10.1186/s12981-022-00456-1

**Published:** 2022-06-26

**Authors:** Ying Wang, Jason W. Mitchell, Chen Zhang, Yu Liu

**Affiliations:** 1 Department of Public Health Sciences, University of Rochester Medical Center, Rochester, NY USA; 2 Department of Health Promotion and Disease Prevention, Stempel College of Public Health and Social Work, Florida International University, Miami, FL USA; 3 School of Nursing, University of Rochester Medical Center, Rochester, NY USA; 4 Division of Epidemiology, Department of Public Health Sciences, University of Rochester Medical Center, 256 Crittenden Blvd, Ste. 3305, Rochester, NY 14642 USA

**Keywords:** Pre-exposure prophylaxis, Intervention, Men who have sex with men, Systematic review, United States

## Abstract

**Background:**

Pre-exposure prophylaxis (PrEP) represents a proven biomedical strategy to prevent HIV transmissions among men who have sex with men (MSM) in the United States (US). Despite the design and implementation of various PrEP-focus interventions in the US, aggregated evidence for enhancing PrEP uptake and adherence is lacking. The objective of this systematic review is to synthesize and evaluate interventions aimed to improve PrEP uptake and adherence among MSM in the US, and identify gaps with opportunities to inform the design and implementation of future PrEP interventions for these priority populations.

**Methods:**

We followed the PRISMA guidelines and conducted a systematic review of articles (published by November 28, 2021) with a focus on PrEP-related interventions by searching multiple databases (PubMed, MEDLINE, Web of Science and PsycINFO). Details of PrEP interventions were characterized based on their socioecological level(s), implementation modalities, and stage(s) of PrEP cascade continuum.

**Results:**

Among the 1363 articles retrieved from multiple databases, 42 interventions identified from 47 publications met the inclusion criteria for this review. Most individual-level interventions were delivered via text messages and/or apps and incorporated personalized elements to tailor the intervention content on participants’ demographic characteristics or HIV risk behaviors. Interpersonal-level interventions often employed peer mentors or social network strategies to enhance PrEP adoption among MSM of minority race. However, few interventions were implemented at the community-, healthcare/institution- or multiple levels.

**Conclusions:**

Interventions that incorporate multiple socioecological levels hold promise to facilitate PrEP adoption and adherence among MSM in the US given their acceptability, feasibility, efficacy and effectiveness. Future PrEP interventions that simultaneously address PrEP-related barriers/facilitators across multiple socioecological levels should be enhanced with a focus to tackle contextual and structural barriers (e.g., social determinants of health, stigma or medical mistrust) at the community- and healthcare/institution-level to effectively promote PrEP use for MSM of color.

**Supplementary Information:**

The online version contains supplementary material available at 10.1186/s12981-022-00456-1.

## Introduction

Men who have sex with men (MSM) experience a disproportionate burden of HIV in the US, accounting for approximately 70% of the 36 thousand new HIV diagnoses in 2019 [1]. Particularly, MSM of color (e.g., Black and Hispanic/Latino MSM) continue to be the priority populations most affected by HIV, representing nearly half of the new infections among all MSM in the US [1–4]. The challenges of HIV prevention in MSM are further complicated by their low perception of HIV risk, the lack of sustainable use of pre-exposure prophylaxis (PrEP), low HIV testing uptake, and high prevalence of behaviors that increase acquisition/transmission of HIV (e.g., condomless receptive/insertive anal sex, multiple anal sex partners, and exchange of sex for money/drugs) [5–7].

Currently approved medications for PrEP, a prescription medicine to prevent HIV infections, include Truvada (for all people at risk for HIV) and Descovy (for people at risk for HIV through anal sex and less impact on kidney and bone health) [8]. When taken as prescribed, daily oral PrEP has been shown to lower the risk of HIV infection from sex by > 99% and from injection drug use by > 74% [9–12]. Mathematical models showed that HIV infections among people who were at high risk for HIV while adhering to PrEP had decreased by 18% from 2016 to 2020 [13]. Therefore, high-impact prevention interventions to enhance PrEP uptake among MSM provide one effective strategy to end the HIV epidemic in the US [14, 15].

PrEP care continuum is usually used to evaluate interventions for PrEP, including (1) awareness (knowledge about PrEP), (2) willingness/intention (likelihood of initiating PrEP), (3) access (linking PrEP candidates to healthcare system), (4) uptake (PrEP initiation), and (5) adherence (adherence to PrEP and retention in PrEP care) [14]. Despite the increased availability and proven efficacy in preventing new HIV infections, the level of engagement along the PrEP care continuum remains low among MSM in the US [5, 16–18]. For example, pooled analyses showed that only 13.9% of MSM have reported ever using PrEP in their lifetime (95% confidence interval (95% CI): 8.8–21.1) [16]. Numerous observational studies with MSM in the US have revealed important barriers across multiple socioecological levels that may affect the uptake/adherence of PrEP, including individual—(e.g., perception of low HIV risk, insufficient PrEP knowledge and concerns over side effects) [19–21], interpersonal—(e.g., lack of parent/peer support) [20–22], healthcare system—(e.g., high cost and low PrEP care quality) [5, 19, 20] and social-cultural—(e.g., stigma, discrimination and medical mistrust) levels [5, 19–21, 23].

Since 2017, there has been an increasing number of interventions to enhance the engagement in PrEP care continuum among MSM by modifying their individual health behaviors or social networks. For example, *PrEPmate* was one of the early mobile health interventions that utilized daily text messages to remind young MSM (YMSM) of PrEP medication [24]. Interventions that leveraged peer influence to improve intentions and willingness to use PrEP among MSM of color were also reported in recent years [25–27]. While these interventions employed novel strategies (e.g., mobile health and social network) and showed efficacy in improving PrEP care continuum in MSM, some limitations were also acknowledged by the authors, including sustainability post intervention period, discrepancy between intervention content and participants’ time-varying intervention needs, and lack of parent/school engagement in PrEP interventions [24–26].

Despite the design and implementation of various PrEP interventions for MSM in the US, there is a need to systematically summarize the practical/theoretical components, modalities, strengths, and limitations of these PrEP-focused HIV prevention interventions for MSM. Aggregated evidence presented on different socioecological levels (e.g., individual, interpersonal, community and healthcare/institution levels)—via a systematic review—enables us to compare interventions across socioecological levels (e.g., acceptability, feasibility or efficacy), informs HIV prevention scientists about successful intervention strategies that modify physical or social environments rather than changing only individual health behaviors, as well as reveals ways to improve current and former PrEP interventions. We conducted a systematic review of intervention studies that aimed to improve one or more aspects of the PrEP care continuum among MSM in the US, by summarizing included studies and their socioecological mechanistic levels, implementation modalities (peer/couple-based, technology-assisted, social network, etc.), and which aspects of the PrEP cascade (e.g., initiation, uptake, and adherence) they targeted.

## Methods

### Literature search strategy

This systematic review was conducted by searching articles via multiple databases (PubMed, MEDLINE, Web of Science and PsycINFO) published by November 28, 2021, following the PRISMA guidelines [28]. The final search terms included: (“gay” OR “men who have sex with men” OR “bisexual” OR “homosexual” OR “homosexuality” OR “same-gender-loving” OR “sexual minority”) AND (“PrEP” OR “pre-exposure prophylaxis” OR “preexposure prophylaxis”) AND (“intervention” OR “trial” OR “experiment” OR “randomized” OR “pre-post”).

### Inclusion/exclusion criteria

Studies were included in this systematic review if they met the following criteria: (1) published journal articles excluding abstracts, conference proceedings, reviews, meta-analyses, editorials or commentaries; (2) conducted in the US; (3) the current and/or the parent study was based on an experimental or quasi-experimental design (e.g., randomized controlled trial (RCT), randomized interventional studies, and pre-post trial) to evaluate the efficacy or effectiveness of a PrEP intervention; (4) reported at least one PrEP care continuum outcome (e.g., awareness, willingness, intention, uptake and adherence); (5) conducted among males who self-identified as gay, bisexual, or reported having sex with men within a past time window; and (6) published in English.

To achieve our goal of comprehensively summarizing PrEP interventions for MSM in the US, we also included the following studies for potential evaluation: (1) studies conducted among MSM and other priority populations (e.g., transgender women); (2) studies that used an experimental design to evaluate the acceptability, feasibility, cost-effectiveness of a PrEP intervention with reporting PrEP-related outcomes; (3) studies using an non-experimental design (e.g., qualitative or cross-sectional study) to assess the acceptability, feasibility or cost-effectiveness of an eligible intervention if details about its implementation to evaluate efficacy/effectiveness could be retrieved from their published parent trials by checking the reference lists; (4) we also included protocols that elaborated the design and implementation to supplement our summary of the PrEP interventions. We excluded studies that used a composite measure of HIV risk with PrEP uptake as one of the risk calculation criteria if PrEP uptake was not explicitly reported. We also excluded papers that described the development/adaptation of eligible interventions without reporting interested PrEP-related outcomes.

### Study screening and data extraction

Titles and abstracts of all identified records were first screened for duplicate removal and relevancy by two independent reviewers (Y.W. and Y.L.). The full text review and data extraction were then conducted independently by one author (Y.W.), and further cross-checked by the other author (Y.L.) for accuracy. Disagreements were resolved by consensus-based discussion. The following information was extracted from eligible studies: study location/setting, study/recruitment period, study design, recruitment strategy, participant characteristics, intervention content, theoretical/conceptual framework, control group, sample size and retention, study outcome measures (e.g., PrEP care continuum outcomes) and major findings from the interventions (e.g., efficacy, effectiveness, feasibility and acceptability).

We categorized all interventions into different socioecological levels based on the primary barriers the interventions aimed to address. The original *socioecological model* to guide HIV studies was composed of four layers: individual, interpersonal, community and structural level [29]. We replaced structural level with healthcare level (i.e., interventions implemented in healthcare settings) since we did not identify interventions that may impact laws or policies. We additionally modified this model by adding a layer of multiple levels to describe interventions that address PrEP-related barriers/facilitators across multiple socioecological levels.

## Results

### Search results

**Fig. 1:**
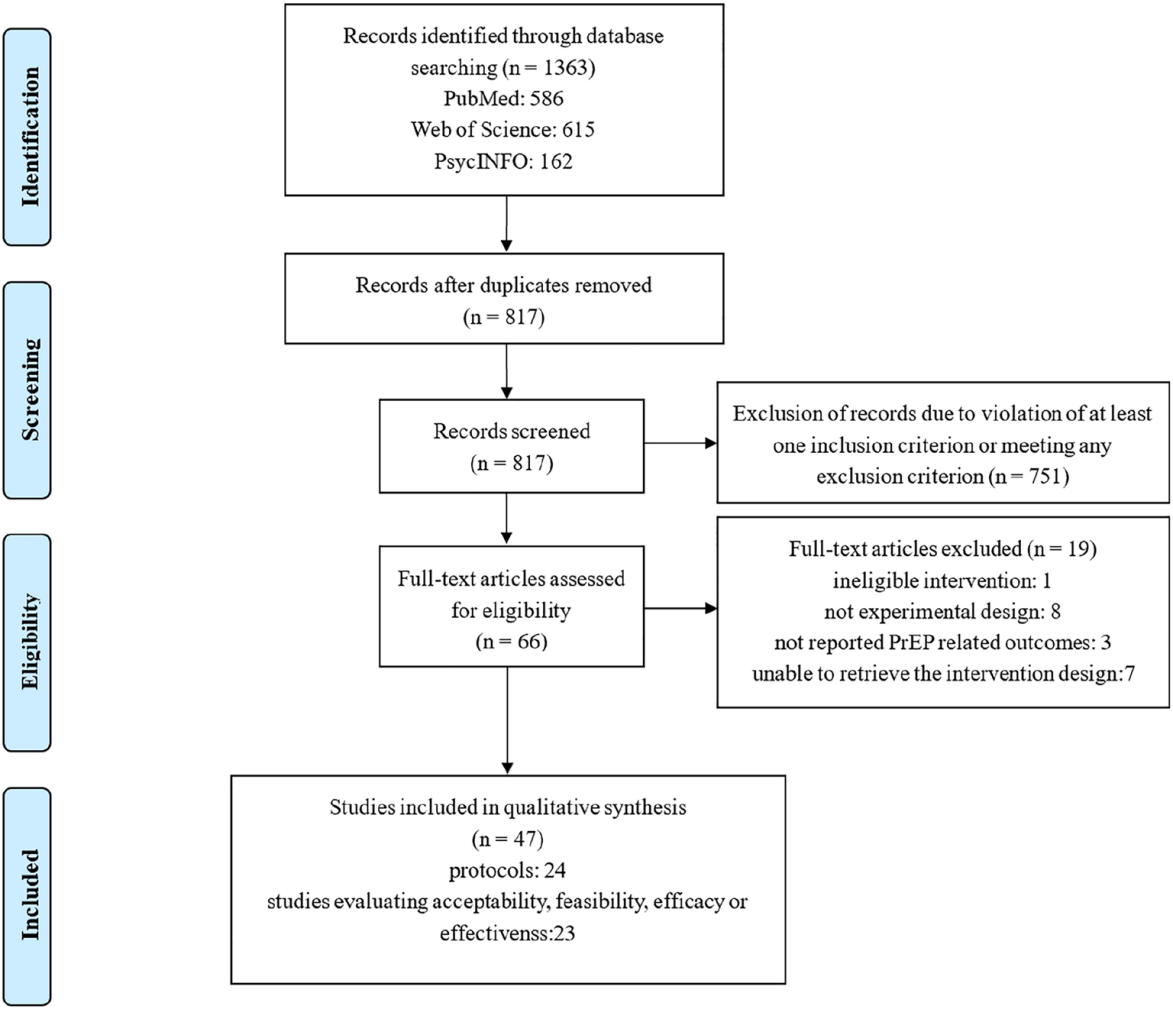
Flowchart of study selection and inclusion procedure

A total of 1363 articles were found through the initial search of multiple databases. After removing duplicates and ineligible articles through title/abstract screening, 66 papers were further assessed via full-text review, with 47 papers representing 42 interventions retained for the final systematic review. Of the included studies, twenty-three papers evaluated the acceptability (n = 8), feasibility (n = 8), efficacy (n = 18) or effectiveness (n = 1) of interventions aimed at improving PrEP uptake and adherence among MSM in the US. Twenty-four of the 47 articles described the protocols for the design and implementation of relevant interventions. Study selection process is shown in Fig. 1.

### Study characteristics

Forty-two interventions were categorized into 5 socioecological levels and characteristics of interventions on each level are presented in Additional file 1: Tables S1–S5, respectively. PrEP interventions for MSM were reported to implement in the US South (Florida, Georgia, Texas, Maryland, Mississippi, North Carolina and D.C.) [30–40], Northeast (Pennsylvania, New York, Massachusetts and Rhode Island) [27, 31–33, 36, 38, 41–49], Midwest (Illinois, Michigan, and Wisconsin) [24–26, 32, 33, 36, 47, 50–52], West (California) [51, 53–58] and nationwide [59–63]. Thirty-seven of the 42 studies focused solely on MSM [24–27, 30–34, 36–41, 43–47, 49–54, 56, 58–72], with some targeting Black, Hispanic/Latino MSM or MSM younger than 34 years old [24–27, 30–34, 36–40, 44–46, 49, 50, 52, 56, 58, 60–63, 70]. Five studies were conducted among MSM and other priority populations(e.g., transgender women or heterosexual men) [35, 42, 48, 55, 57]. A RCT design was used to evaluate 37 interventions on their effect on PrEP-related outcomes [24, 25, 27, 30–42, 44–48, 50, 52–56, 58–63, 66–72], whereas 5 interventions were evaluated using a quasi-experimental design [26, 43, 49, 51, 57] and 4 via a pretest-posttest design [26, 49, 51, 57].

### Study outcomes across pre-exposure prophylaxis care continuum

Most interventions were evaluated by PrEP-related outcomes across the PrEP care continuum with PrEP uptake (n = 29) and adherence (n = 24) most commonly reported. Awareness of PrEP was measured by participants’ knowledge about PrEP including medication purpose, side effects and self-efficacy [25–27, 31, 33, 39, 40, 43–45, 49, 56, 60, 63, 70]. PrEP willingness/intention focused on participants’ attitudes towards PrEP use such as their likelihood of initiating PrEP across various conditions or during a future time window (e.g., within the next 3 or 6 months) [25–27, 31, 33, 34, 39, 44, 57, 59, 60, 63, 70]. Access to PrEP was referred to participants’ linkage to healthcare system (e.g., scheduling or attending an appointment for PrEP consultation) [25, 27, 33–35, 39, 52, 58, 69–71]. PrEP uptake was measured by self-reported PrEP initiation or a recipient of a prescription for PrEP [25, 30, 31, 34–36, 38, 39, 43–46, 52, 54, 55, 57, 58, 60, 69–72]. Adherence to PrEP utilized both behavioral (e.g., self-reported retention in PrEP care or number of doses missed in the past 30 days) [30–33, 36, 40, 46, 48–51, 58, 60, 73] or biological measurements (e.g., PrEP concentration in dried blood spots) [24, 32, 33, 37, 40–42, 53, 72]. Outcomes out of the PrEP care continuum were also reported in a few studies, including descriptive and subjective PrEP norms, PrEP-related stigma and barriers to PrEP [25–27].

### Intervention strategies and findings

Building on the *Social-ecological Model* and the characteristics of the reviewed interventions [29], we categorized 42 interventions into individual-, interpersonal-, community-, healthcare/institution-level, and multilevel interventions. The vast majority of these interventions were delivered at the individual or interpersonal level. The sub-categories under individual-level interventions were not mutually exclusive (e.g., a technology-assisted intervention may include personalized/individualized elements). Hence, the intervention types/levels presented below were used to provide the audience with examples of various interventions.

### Individual-level interventions

#### PrEP regimen interventions

*PrEPare* was one of the initial intervention trials that tested the effect of daily tablets combined with a behavioral intervention on adherence to PrEP in YMSM compared to placebo pill control combined with the behavioral intervention and behavioral intervention alone [50]. Another trial implemented in New York City evaluated 3 different dosing regimens of PrEP, 1 tablet twice weekly with a post-sex dose, 1 tablet before and after sex and 1 tablet daily [42]. Both interventions suggest that daily oral PrEP was associated with a high level of medication adherence compared to other dosing recommendations [42, 50].

#### Technology-assisted interventions

In recent years, text messages have become one of the most indispensable components used in PrEP interventions [33, 52]. For example, participants in *LifeSteps* received text messages on a weekly basis as a motivational reminder to enhance PrEP adherence [33]. Using the *Behavioral Theories* and *Information-Motivation-Behavioral Skills* (IMB) framework, bidirectional text message interventions were implemented to boost communication between participants and research team [24, 52, 53]. For example, daily pill reminder messages were sent to participants at a personally selected time, with study staff providing assistance to participants who did not respond to the message or reported any difficulty with PrEP use [24, 51, 53]. *Partner Services PrEP* study also used text messages to deliver booster sessions to follow up on participants’ experiences getting linked to PrEP medication [52]. Overall, text message interventions based on mobile technology were found to be feasible, acceptable and efficacious when the messages were personalized and able to address specific needs of the target population [24, 51, 53].

The development of app-based interventions addressing individual level barriers/facilitators has proliferated since 2017. Most apps were grounded in health behavior change theories, such as *Social Cognitive Theory* (SCT), *Social Learning Theory* and the IMB model [30, 32, 40, 45, 47, 61, 70]. The apps compiled relevant information that may influence PrEP use (e.g., education of correct HIV risk perception, medication efficacy, self-efficacy and social norms) and integrated supports from local PrEP providers to improve participants’ awareness of PrEP and facilitate linkage to PrEP care [30, 37, 45, 47, 61, 62, 70]. Other key features that might facilitate participants’ adherence to PrEP included medication reminder, graphical tracking of medication adherence and day-to-day strategies to counter relevant barriers (e.g., PrEP stigma) [32, 49]. App-based interventions were found to be acceptable and feasible [57, 64, 65], but their efficacy remains unclear as most studies are still ongoing [32, 37, 47, 61, 62, 70, 72].

Of particular note, game-based interventions have been gaining popularity in recent years. For example, *Viral Combat* is one of the early apps that used gamification to increase adherence to PrEP among YMSM. In this game, players gain points by engaging with healthcare providers (HCPs), initiating and adhering to the PrEP medication [40]. This game demonstrated that intervention participants were 3.75 times more likely to engage in optimal PrEP dosing compared to those who received a non-PrEP related mobile game (95% CI 1.20–11.77).

Other technology-based interventions at the individual level included interventional videos or messages delivered through open network social media platforms/websites (e.g., Facebook, Instagram, Reddit, Twitter) [44, 59, 63]. For example, van den Berg et al. examined whether SCT-based and culturally congruent social media messages would increase PrEP knowledge among Black and Hispanic MSM [44]. This trial is ongoing and the results have not yet been reported.

#### Personalized interventions

Some technology-assisted interventions often incorporate personalized/individualized elements by customizing the intervention content based on participants’ demographic characteristics or HIV risk behaviors [31, 44, 47, 49, 62]. For example, *M-Cubed* intervention tailored HIV prevention messages to participants’ self-reported HIV status and level of HIV risk [47]. In an intervention for MSM of color, cognitive interviewing was used to develop HIV prevention information tailored to participants’ serostatus and culture [44]. Most personalized interventions are ongoing and have not reported their results [31, 44, 47, 62].

#### Other individual-level intervention strategies

The *PrEPARE2* intervention tested whether provision of objective HIV risk score to MSM had a positive impact on their uptake of PrEP [54]. The score was generated from a mathematic model that considered both HIV risk behavior (e.g., condomless anal intercourse) and biological outcomes [e.g., sexually transmitted infections (STIs)]. It is reported that *PrEPARE2* did not increase PrEP initiation among MSM (11% vs. 10%, p > 0.99) [54].

### Interpersonal-level interventions

#### Overall peer-based interventions

Most peer-based interventions utilized peer interventionists to enhance engagement of minority MSM in the PrEP care continuum. In an ongoing peer-navigation intervention, Spanish–English bilingual peer lay navigators delivered PrEP-focused educational modules to Latino MSM [58]. In a different intervention that utilized enhanced PrEP adherence support, peer navigators led both in-person and online groups to provide adherence support to Black MSM [48]. However, favorable changes in self-reported PrEP adherence were not observed for this enhanced adherence intervention [48].

We also observed that peer-based interventions often incorporated personalized elements [34, 38, 55, 56]. In a culturally-tailored counseling intervention, Black MSM interventionists helped participants identify and address their barriers to PrEP initiation (e.g., health insurance, mental health violence, alcohol and substance abuse) based on their prevention needs, and referred them to appropriate prevention resources [34]. This counseling intervention demonstrated preliminary efficacy where 24% of participants in the intervention group initiated PrEP compared to no one in the control group (p = 0.02) [34].

*Motivational interviewing* (MI) was another common strategy applied in peer-based interventions. Peer mentors would use MI to help MSM resolve their ambivalence about behavioral change as they moved through the different stages of change (e.g., contemplation, determination and action) [38, 55, 56]. In an ongoing coach-based, mobile-enhanced intervention, participants who reported barriers to telephonic engagement in HIV prevention services would be connected with peer coaches, who would empathize with them and assist by exploring alternative means to help retain them in the study [38].

#### Couples-based intervention

We identified one young male couple-based intervention, *We Prevent* [60]. Guided by relationship-oriented IMB model, *We Prevent* aimed to enhance MSM-specific sexual health knowledge (e.g., risk within dyads), motivation (e.g., peer norms towards HIV prevention in relationships) and HIV risk-reduction skills (e.g., couples HIV testing and counseling and PrEP) via two sessions delivered to male couples. The intervention also employed MI techniques to teach identification of unhealthy relationships and communication strategies with partners to help them prepare for engaging in HIV prevention services as a couple [60]. This couples-based intervention is ongoing and its efficacy is unknown.

#### Social network interventions

Based on the framework of IMB and SCT, three social network interventions, *E-PrEP*, *PrEP Chicago* and one conducted in Wisconsin, were developed for young Black or Latino MSM [25–27]. These interventions focused on improving participants’ knowledge about PrEP and subsequent PrEP initiation, and leveraged peer influence to scale up PrEP uptake in peers’ social networks. Participants in the *E-PrEP* intervention posted targeted materials on social media to provide PrEP education to peers in their existing online networks [27]. The other two peer change agent-based interventions emphasized training of communication skills and conversational strategies to ensure peer change agents could advocate PrEP use effectively. Peer change agents learned how to address their friends’ concerns about PrEP (e.g., sigma, misconception, effectiveness and side effects) while also engaging with them to help facilitate development of positive attitudes toward PrEP [25, 26]. Social network interventions exhibited high acceptability and efficacy in improving PrEP knowledge, attitudes, and self-efficacy among young minority MSM [26, 68].

### Community-level interventions

An active PrEP patient navigation was one of the few interventions that leveraged community engagement in HIV prevention programs [35]. Guided by the model of community-based case management that focused on utilization of support and resources in the community, patient navigators assisted participants with overcoming barriers to PrEP linkage and identifying available sources of support in the community [35]. For example, if participants reported administrative costs (e.g., notary services) as a barrier to PrEP initiation, patient navigators would then provide them with information on related community services free of charge. The community-level intervention showed preliminary efficacy to facilitate PrEP initiation, yet no significant differences existed between the intervention and control groups [35].

### Healthcare/institution-level interventions

Interventions at the healthcare/institution level usually involved HCP for PrEP promotion in various healthcare settings. In most healthcare-level interventions, HCPs (e.g., nurses, STI clinic counselors) provided participants with information on PrEP access, used MI to encourage PrEP initiation, or taught behavioral skills to address PrEP-related barriers (e.g., PrEP stigma coping) [36, 41, 43, 69]. *Life-Steps for PrEP* and one brief behavioral intervention additionally offered booster sessions to construct/refine medication adherence plans and monitor participants’ long-term adherence to PrEP [41, 69]. Healthcare-level interventions were found to be efficacious for improving PrEP awareness, PrEP appointment scheduling, and PrEP initiation among MSM [43, 69]. However, significant differences for PrEP adherence, via measurement by an electronic pill storage device, were not observed in the *Life-Steps for PrEP* intervention [41].

### Multilevel interventions

Interventions that address PrEP determinants across multiple socioecological levels represent an important interventional mechanism to effectively promote PrEP use among MSM [31, 57]. One of the few examples was *Get Connected*, a web app-based intervention guided by *Integrated Behavioral Model* and *Self-Determination Theory*, that combined both individual- and healthcare-level strategies to help YMSM overcome multiple barriers to PrEP care [31]. At the individual level, the app delivered personalized educational materials to participants to increase their awareness of HIV risk and self-efficacy for HIV prevention. At the healthcare/institution level, participants were asked to rate the clinic where they got tested for HIV or received PrEP evaluation. The assessment used a composite measure which took into account the clinical environment, service quality, privacy/confidentiality, perceived provider competency, etc. Participants’ evaluations were sent to sites to help them understand and improve their performance that may benefit their future PrEP clients [31]. This app is still being tested and the results have not been reported.

*We Are Family* represents another multilevel intervention conducted in San Francisco [57]. At the individual level, information on HIV prevention and local prevention resources were delivered to participants through in-person group sessions and the *We Are Family* app. Participants were allowed to support and connect with each other by sharing their stories of battling HIV-related stigma. The research team also hosted or sponsored community-level events such as prevention balls, game nights and holiday parties to leverage community norms to facilitate PrEP uptake in MSM. At the healthcare level, a healthcare provider worked with the community to provide HIV prevention services including HIV testing or PrEP referral. This multilevel intervention was found to be acceptable, feasible, and demonstrated preliminary efficacy in facilitating PrEP intention, initiation and adherence among MSM [57].

## Discussion

The present systematic review provides a concise, informative summary of what PrEP-related interventions have occurred with MSM in the US. Most individual-level PrEP interventions were technology-assisted and delivered via messaging platforms and/or apps. Compared with traditional venue-based interventions, technologically delivered PrEP interventions are convenient, cost-effective, and may help overcome system-level barriers to PrEP care (e.g., transportation to clinics and inconvenient clinic locations and hours) [74, 75]. In addition, app-based interventions included in this review provided extensive information on PrEP, ranging from medication effectiveness, side effects, self-efficacy to local PrEP resources—all aimed to improve participants’ awareness of PrEP and help them build behavioral skills to use PrEP [30, 32, 45, 47, 61, 70]. Some of the app-based interventions included interactive features (e.g., quizzes, exercises, discussions and games) to facilitate participants’ continued use of the app (i.e., engagement) [32, 57, 61]. In contrast, text message interventions contained fewer interactive features. One key strength of text message interventions centered on them requiring fewer resources for development and pilot testing (vs. apps). In general, text message interventions were found to be high acceptable by MSM [24, 48, 51, 53]. Further, participants could tailor for when and how often text messages would be sent to accommodate their schedules for daily PrEP intake [24, 51, 53]. Bidirectional messages may provide researchers with opportunities to better understand participants’ medication adherence patterns and identify when to provide assistance when necessary [24, 51, 53]. These findings suggest text messaging interventions have the potential to retain MSM in PrEP care.

It is also important to acknowledge the gaps in current technology-assisted interventions. Development of apps and platforms for interventions remain a barrier [51, 65]. Commonly reported issues regarding app design and functions included lack of diversity in the presentation of educational information, inability to link social media profiles, lack of common functions (e.g., customizable reminders), and technical glitches (e.g., slow responsiveness and app crashing) [30, 45, 51]. Therefore, formative studies to learn participants’ preference regarding the app design are necessary to ensure the successful implementation of technology-based interventions. In addition, there might be a mismatch between intervention content and time-evolving prevention needs of MSM [24, 51]. For example, some visual/textual components used in an intervention might only offer introductory information skills to facilitate PrEP initiation, which would be less useful to experienced PrEP users with challenges with PrEP adherence [24]. To meet the evolving needs of MSM, future PrEP interventions could be designed and tailored to better align with the PrEP continuum and men’s ongoing needs.

We observed that approximately 60% of the existing PrEP interventions were designed for YMSM; and more than 70% of these interventions relied on technology to facilitate PrEP uptake. This finding aligns with the ever-growing efforts in recent years to address the elevated HIV epidemic among YMSM in the US [4]. Given the low rates of PrEP use among YMSM and preference of intervention modality supported by technology (e.g., social media, networking apps, internet) [76, 77], technology-assisted interventional components may bode well in future PrEP interventions to enhance the PrEP care continuum among YMSM [24, 49, 52, 66].

Several interpersonal-level PrEP interventions designed and implemented among YMSM (e.g., peer-deliver/navigation, couple-based, social network-based interventions) met inclusion criteria for the present review. However, interpersonal interventions that involve parent or school educators to promote PrEP among YMSM are notably missing from the current review. Substantial evidence indicates parent’s low level of PrEP awareness, perceived HIV and LGBTQ+ related stigma and negative reaction to PrEP, along with adolescents’ poor self-efficacy to communicate with parents about PrEP and/or sexual orientation were all reported barriers to PrEP use among YMSM [22, 78, 79]. Interventions that address parental negative attitudes towards PrEP and sexuality, and that also promote parent-adolescent communication may hold promise to enhance PrEP uptake and adherence among YMSM. None of the included interventions were implemented in school settings or educational agencies. As adolescents and YMSM may spend a majority of their day at school, the development and implementation of contextually appropriate interventions at schools, by involving trusted school-based peers/counselors, may offer unique opportunities to provide education about PrEP and HIV prevention among these priority populations.

Black and Hispanic/Latino MSM (i.e., MSM of color) were also priority populations in the included PrEP interventions, given their heightened HIV burden and low rates of PrEP uptake [2, 3, 80, 81]. Findings from the present review revealed that some of the most important strategies in PrEP interventions for MSM of color was the utilization of peer influence, which led to increased cultural congruence, reduced PrEP/HIV-related stigma, facilitated trust/access to PrEP care, and motivated conformity to peer norms/behaviors (i.e., social comparison) to promote PrEP initiation/adherence [25–27, 34, 38, 48, 56, 58].

However, challenges remain for PrEP interventions for MSM of color. First, MSM of color may be less likely to participate in online HIV interventions given the racial/ethnic disparities in the use of technology for health-related purposes [82, 83]. For example, almost all Black MSM completed the face-to-face session in *Partner Services PrEP* study, while only a limited number of participants completed the booster session delivered via mobile phone [52]. Second, our aggregated evidence reflects the lack of interventions to tackle community-level determinants of PrEP care for MSM of color. Community-level interventions that address broader contextual and structural issues by improving social determinants of health (e.g., neighborhood environment, housing and food insecurity) should be further strengthened [48, 84]. Last, we identified only one intervention, *Get Connected*, that overcame system- and structural-level barriers to PrEP (e.g., stigma and medical mistrust) by providing HIV care that is sensitive and inclusive to MSM of color. Interventions delivered to HCPs to enhance clinical experiences of MSM of color are still missing from the current literature.

We identified four interventions implemented at the healthcare/institution level. All these interventions involved educational modules delivered by HCPs in HIV/STI clinics [36, 41, 43, 69]. One of the gaps in the healthcare/institution-level interventions is the lack of follow-up for long-term adherence to PrEP. We observed improvement in behaviors that align with the earlier stages of the PrEP continuum (e.g., increased PrEP awareness, scheduling and attending a PrEP appointment, and initiating PrEP care) [43, 69], but significant differences were not observed for long-term adherence to PrEP [41]. Current models of care in HIV/STI clinics primarily provide STI and HIV testing services and are not well suited for transitioning to a longitudinal model of HIV prevention/care due to the absence of protocols to guide clinical practice and low capacity of trained HCPs to provide PrEP care [73]. One potential solution is to provide training to PrEP counselors, who would then be responsible for monitoring PrEP use among those who have initiated the medication and providing follow-up counseling services. Another gap is the lack of culturally trained primary healthcare providers (PCPs) with specialty in HIV/STI prevention/care for sexual and gender minority populations. Although HIV/STI clinics are ideal settings to reach populations who are at elevated risk for HIV infection [85], PCPs as the first point of contact into healthcare have a unique opportunity to reach the majority of patients who are less aware of PrEP and may be in need of this preventative medication. For example, evidence from an HIV prevention program in Washington State showed a pronounced increase in uptake of HIV prevention services (e.g., HIV testing) among MSM who received healthcare from providers that were not from HIV/STI clinics, suggesting the great potential of the entire healthcare system rather than only HIV/STI care providers in promoting uptake of HIV prevention services [86]. Additionally, the prescription of PrEP in primary healthcare settings, where the primary purpose is not HIV prevention and care, may have the potential to reduce stigma surrounding HIV among MSM and thereby may help promote PrEP acceptance [73]. However, barriers such as insufficient PrEP knowledge and lack of skills/motivation to discuss PrEP with MSM clients must be addressed among PCPs before effective structural/institution interventions can be implemented [87–89].

Our study is among the few that have systematically summarized and evaluated PrEP interventions for MSM in the US to inform the design and implementation of future interventions. There are also limitations to this review. First, the literature search strategy may be not comprehensive and thus we were unable to incorporate all relevant interventions into this systematic review. Second, the categorization of interventions into each socioecological level was based on the primary barriers the interventions aimed to address. Therefore, the intervention levels presented in this review may be not precise and were used to provide examples of interventions across socioecological levels only. Third, more than 40% of the studies are ongoing. Their effect on promoting PrEP uptake and adherence is unknown. However, our primary objective is to summarize the practical/theoretical components, modalities, strengths, and limitations of these studies to inform the design of future PrEP interventions. In addition, some studies with small sample size may have low statistical power [26, 34, 35, 41, 49–51, 54]. Scaled-up RCTs as well as intervention assessment in real-world settings are further required to replicate their results. Finally, this systematic review identifies the following research opportunities based on the gaps in existing studies: (1) expanding the spectrum of participants (e.g., MSM with injection drug use) given their elevated risk for HIV; (2) testing the effect of different regimens of PrEP (e.g., daily oral pill vs. long-acting injectable medication) on PrEP care continuum engagement given the evidence that reduced pill burden may increase PrEP uptake/adherence among MSM [42, 50]; and (3) taking into account different brands of PrEP when designing and evaluating interventions in light of the recent approval of Descovy for PrEP.

## Conclusions

Low level of PrEP uptake and medication adherence among MSM, especially young and/or those of color, is concerning. Interventions to improve PrEP uptake and adherence among MSM have been designed and implemented at multiple socioecological levels (e.g., individual, interpersonal, community and healthcare/institution) in recent years, with many of them currently in progress via a RCT. Fully evaluated interventions, as well as those currently in progress, may hold promise to help facilitate PrEP adoption among MSM; mechanisms used to help improve one or more stages of the PrEP continuum were also noted in the included interventions. Areas for improvement were identified and were presented as future research opportunities to improve current and future PrEP interventions for MSM in the US.

## Supplementary Information

**Additional file 1: Table S1.** Summaryof study characteristics: individual-level interventions. **Table S2.** Summary of study characteristics: interpersonal-levelinterventions. **Table S3.** Summary ofstudy characteristics: community-level interventions. **Table S4.** Summary of study characteristics: healthcare system-levelinterventions. **Table S5.** Summary ofstudy characteristics: multilevel interventions.

## Data Availability

All data generated or analysed during this study are included in this published article and its Additional files.
