# Supplementary material for: Evidence and implication of interventions across various socioecological levels to address pre-exposure prophylaxis uptake and adherence among men who have sex with men in the United States: a systematic review
Source: AIDS Res Ther. 2022 Jun 26;19:28. doi: 10.1186/s12981-022-00456-1 (PMC9233830; doi:10.1186/s12981-022-00456-1)
Supplement: Supplementary file 1 — Additional file 1: Table S1. Summaryof study characteristics: individual-level interventions. Table S2. Summary of study characteristics: interpersonal-levelinterventions. Table S3. Summary ofstudy characteristics: community-level interventions. Table S4. Summary of study characteristics: healthcare system-levelinterventions. Table S5. Summary ofstudy characteristics: multilevel interventions. [file 12981_2022_456_MOESM1_ESM.docx]

Table S1. Summary of study characteristics: individual-level interventions

| Source | Location/setting | Recruitment/Study period | Study design | Recruitment strategy | Population characteristics | Intervention (components) | Theoretical/conceptual framework | Control | Sample size & retention (intervention vs. control) | Outcome measures | Findings (acceptability, feasibility, efficacy or cost-effectiveness) |
| --- | --- | --- | --- | --- | --- | --- | --- | --- | --- | --- | --- |
| Hosek et al. (2013) [50] | Chicago, IL | Not reported | RCT | Participants were approached by study staff at community-based agencies and youth venues. | Young MSM  Age (mean±SD): 19.97±1.3  Black/African American: 53%  Latino: 40% | “PrEPare” (ATN082):  Daily co-formulated emtricitabine (FTC) and tenofovir disoproxil fumarate (TDF) as PrEP | Not reported | (1) placebo pill control; and (2) “no pill” control. | 58 Participants were randomized (20 vs. 19 vs. 19) and they followed for 24 weeks with follow-up assessments conducted at 0, 4, 8, 12, 16, 20 and 24 weeks. Retention to study visits was 98.5% overall. | Adherence to study drug evaluated by Time-Line Follow Back | (1) The feasibility of enrolling at risk youth, particularly MSM of color, into Project PrEPare were demonstrated.  (2) The acceptability of the intervention along with counseling and testing was high.  (3) Rates of detectable tenofovir in plasma of participants in the FTC/TDF arm ranged from 63.2% (week 4) to 20% (week 24). |
| Mustanski et al. (2014) [59] | Nationwide | June 6 to June 20, 2012 | RCT | Banner ads placed on Facebook | MSM aged 18 years and older  White: 77.5%  Hispanic/Latino: 14.8%  Age (median): 23 (IQR:13)  Homosexual/Gay: 95.1% | Combination messages intervention:  Four HIV prevention videos were developed whose sole  focus was either about condoms, PrEP, nPEP, or rectal microbicides for HIV prevention. Participants assigned to the intervention group viewed informational messages about two, or four of the prevention videos. | Not reported | Participants viewed informational messages about one of the four prevention options. | One vs. two vs. four message group: 404 vs. 313 vs. 86 | Intention to use PrEP: “If a doctor were to prescribe PrEP to you in the next 12 months based on your pattern of sexual risk, how likely would you be to use PrEP to prevent HIV?” | Efficacy (one vs. two vs. four message group):  Mean intention: 2.86 vs. 3.50 vs. 3.65 (p<0.05) |
| Acceptability evaluation: Sullivan et al. (2017) [65] | Atlanta, GA; Jackson, MS; and Washington, DC | Launched in January 2020 | RCT | Online venues including social networking sites (eg, Facebook, Instagram, and Twitter), sexual networking apps (eg, Grindr and Scruff), and banner ads on websites frequented by MSM (eg, Queerty, Towleroad, and Adam for Adam); flyers; and recruitment through community partners (eg, community-based organizations and drop-in centers | MSM aged 18 to 34 years | “HealthMindr”: a mobile app-based intervention  Participants will receive access to the full HealthMindr app, with information and resources about PrEP, other HIV prevention information, ability to order free HIV/sexually transmitted infection test kits, and additional resources related to substance use and mental health. | Social cognitive theory | Participants in the control arm will use the HealthMindr app but will only have access to the study timeline and a message center to communicate with study staff. | Participants will be followed for 12 months with follow-up assessments conducted at 3, 6, 9 and 12 months. | (1) Rate of PrEP uptake as measured by self-report in the app-based surveys confirmed by laboratory testing or uploading a photograph of their PrEP prescription or pill bottle; (2) self-reported adherence: number of doses missed in the past 30 days; and (3) persistence: time on PrEP | Usability and acceptability:  (1) Eight of 86 (9%) PrEP-eligible MSM started PrEP during the 4-month period; of those, 6 of the 8 reported that the app influenced their decision to start PrEP.  (2) System usability scale: 73 (above average). |
| Protocol: Jones et al. (2020) [30] |  |  |  |  |  |  |  |  |  |  |  |
| Fuchs et al. (2018) [51] | San Francisco, CA; and Chicago, IL | Not reported | Pretest-posttest | Participants were recruited from iPrEx OLE Study. | HIV negative MSM taking PrEP  Age (range): 21–66  White: 67.9%  Black: 12.5% | “iText”: a tailored mobile health intervention  The intervention utilized weekly bidirectional text or e-mail support messages to encourage PrEP adherence. Participants can personalize both the content and timing of the messages. | Not reported | Pretest number of days doses were missed | 52 participants completed the 12-week pilot study. | PrEP adherence: (1) self-reported number of days doses were missed in the previous 30 days; and (2) the number of doses missed in each reporting period as measured by clinic-based pill counts | Acceptability:  56% reported that the messaging strategy was helpful.  Adherence effects:  Relative risk reduction in missed doses before and after intervention (95% CI):  (1) Self-report: 0.45 (0.19–1.06)  (2) Days missed as measured by pill counts: 0.23 (0.08–0.67) |
| Grant et al. (2018) [42] | Harlem in New York City | July 2012 to May 2014 | RCT | Participants were recruited from a community clinic and clinical research site. | MSM (97%) and transgender women  Age 18–24: 30%  Age 25–29: 18%  Age 30–39: 21%  Age >=40: 31%  Black: 70%  White: 13%  Hispanic: 25% | HPTN 067/ADAPT: Participants were randomly assigned to: (1) 1table daily; (2) 1 tablet twice weekly with a postsex dose (time-driven), or (3) 1 tablet before and after sex (event-driven). | Not reported | Participants were randomly assigned to 1 tablet daily. | Daily vs. time-driven vs. event-driven  Baseline: 59 vs. 60 vs. 60  Week 10: 57 vs. 57 vs. 56  Week 14: 54 vs. 51 vs. 54  Week 18: 53 vs. 49 vs. 53  Week 22: 50 vs. 50 vs. 51  Week 26: 50 vs. 51 vs. 49  Week 30: 49 vs. 50 vs. 49  Week 34: 45 vs. 49 vs. 47 | (1) Self-reported coverage of anal and neovaginal intercourse events with pre- and postexposure dosing of PrEP defined as at least 1 tablet reported taken within 96 hours prior to intercourse and another tablet reported taken within 24 hours after intercourse  (2) Tenofovir diphosphate (TFV-DP) concentrations in dried blood spots (DBS) suggested use of ≥2 tablets on visits when sex was reported in the prior week | Daily vs. time-driven vs. event-driven:  (1) Self-reported complete coverage:  66% vs. 47% (P = 0.01 vs. daily) vs. 52% (P = 0.01 vs. daily)  (2) DBS analysis: 48.5% vs. 30.9% (P = .11 vs. daily) vs. 16.7% (P = .004 vs. daily) |
| LeGrand et al. (2018) [32] | Bronx, NY; Chicago, IL; Atlanta, GA; Houston, TX; Boston, MA; and Philadelphia, PA | Anticipated to begin in winter 2018 | RCT | In-person, venue-based (including recruitment from local PrEP clinics or providers), and Web-based recruitment | Young MSM aged 16 to 24 years | “P3 (Prepared, Protected, emPowered)”: a mobile app-based intervention  (1)P3: The app includes social features, a PrEP-specific knowledge center, game elements, loss aversion mechanics, and a daily habit-building interface.  (2)P3+: This version of the app will include all features of the P3 app as well as two-way text messaging sessions with trained counselors. | Social cognitive theory, narrative communication (eg, storytelling), principles of persuasive technology, and Fogg behavioral model of persuasive technology | Standard of care will be available to participants at their prescribing PrEP provider’s practice and they will be provided with written materials regarding PrEP adherence. | Participants will be followed for 6 months with follow-up assessments conducted at 3 and 6 months. | (1) PrEP adherence, measured by the levels of TFV-DP and emtricitabine-triphosphate (FTC-TP) in DBS plasma, consistent with >4 doses per week; (2) self-reported retention in PrEP clinical care; and (3) PrEP persistence | The trial is ongoing. |
| Moore et al. (2018) [53] | Southern California | February 2013 to February 2016 | RCT | Participants were recruited from 4 Southern California medical centers (University of California, San Diego; University of Southern California; Harbor–University of California Los Angeles; and Long Beach Health Department)。 | MSM only  Age (mean): 35.2  Non-Hispanic white: 50%  Non-black Hispanic: 28%  Black alone or as part of multiple racial identity: 15% | “iTAB”: a personalized, 2-way, automated text-messaging intervention  On a daily basis, participants received a mix of health promotion and “factoid” messages at a personally selected time consistent with when they planned to take PrEP. Participants worked with study staff to choose messages consistent with their preferences from 440 different messages. Participants could also create their own messages. | Behavioral theory | Standard of care: Participants received brief HIV prevention and adherence counseling with provision of study drug | Baseline: 200 vs. 198  Week 48: 324 (81.4%) participants were still on study | (1) Adequate adherence: DBS TFV-DP concentrations at both week 12 and 24 of >719 fmol/punch; (2) near-perfect adherence: DBS TFV-DP concentrations of >1246 fmol/punch and (3) dosing within the past 24 hours: plasma FTC >350 ng/mL | (1) Adequate adherence: 72.0% vs. 69.2%, p > 0.05  (2) near-perfect adherence: 33.5% vs. 24.8%, P = 0.06  (3) dosing within the past 24 hours at week 12: 47.5% vs. 33.3%, P = 0.007. This difference did not persist in week 24, 36, or 48. |
| van den Berg et al. (2018) [44] | Rhode Island; and Bristol County, MA | Anticipated to begin in August 2019 | RCT | Ads online and on public transportation; posted signs and flyers in local community and commercial venues; and in-person outreach at places where the target population congregates, such as community-based organizations, clinics, bars, and clubs | HIV-positive and HIV-negative Black and Hispanic MSM | A culturally tailored intervention:  Participants will receive guided social media messages plus the newly developed website. The website and messages to be tailored to participant serostatus and race/ethnicity | Information motivation behavior skills model and social cognitive theory | Participants have access to website only. | Participants will be followed for 6 months, with follow-up assessments collected at 6 months. | (1) Levels of and increases in TasP or PrEP uptake; and (2) increases in levels of TasP- and PrEP-specific knowledge, favorable attitudes, and behavioral intentions regarding TasP and PrEP | The trial is ongoing. |
| Pilot trial: Wray et al. (2018) [67] | Northeastern US | Launched in January 2019 | RCT | Gay-oriented smartphone dating apps (e.g., Grindr and Scruff); social networking sites (e.g., Facebook and Instagram); and in-person outreach (e.g., flyers) | High-risk MSM only | (1) “eTEST”: a mobile app-based intervention  Participants received HIV self-testing kits equipped with devices that detected when kits were opened. Within 24 h of opening the kit, a counselor will call participants to conduct post-test counseling and refer them to other needed services, including PrEP  (2) standard HIV self-testing kits with no follow-up | Not reported | Text messages will be sent to participants once every 3 months to remind them to get tested for HIV in a local clinic and provide them with information about free clinic-based testing in the area. | Participants will be followed for 12 months, with follow-up assessments collected at baseline, 1, 4, 7, 10 and 12 months. | (1) The proportion of participants who consulted with a physician about PrEP, (2) received a PrEP prescription | Preliminary efficacy:  (1) eTEST participants were more likely to receive PrEP referrals than then other conditions (p < 0.05).  (2) Slightly higher and nonsignificant rates of having been prescribed PrEP among eTEST participants than the other conditions |
| Protocol: Wray et al. (2020) [71] |  |  |  |  |  |  |  |  |  |  |  |
| Biello et al. (2019) [33] | Atlanta, GA; Boston, MA; and Chicago, IL | Anticipated to begin in early 2019 | RCT | Active recruitment at study sites, local organizations and venues that young MSM attend; posting study information through flyers, posters, and palm cards at these venues; banner ads on popular Web-based social media outlets | Young MSM aged 15 to 24 years | “LifeSteps for PrEP for young MSM”:  The intervention includes (1) 4 sessions (medication adherence, sexual behavior, and problem solving to overcome barriers to adherence); and (2) weekly SMS text messaging to support adherence, as well as to understand participants’ patterns of behavior. | Gelberg-Andersen behavioral model for vulnerable populations and motivational interviewing techniques | PrEP standard of care | Participants will be followed for 6 months, with follow-up assessments collected at baseline, 3 and 6 monthsIRA | (1) Biological correlates of adherence: DBS drug levels of tenofovir diphosphate and emtricitabine triphosphate; (2) self-reported PrEP adherence assessed using timeline follow back; (3) medical appointment adherence: proportion of scheduled clinic visits attended by each patient; (4) readiness to use PrEP: how likely they are to use or continue using PrEP under a variety of circumstances; (5) behavioral skills for PrEP use: how “hard” or “easy” it was for participants to implement a variety of skills, including discussing side effects with medical providers and remembering to take pills on time; and (6) PrEP taking self-efficacy: confidence to take medications in various situations | The trial is ongoing. |
| Protocol: Biello et al. (2019) [45] | Boston, MA, and Bronx, NY | Launched in October 2018 | RCT | Recruitment at organizations and venues where MSM attend; flyers, posters, and palm cards at these venues; advertisements on popular Web-based social media outlets (eg, Facebook, Grindr, etc). | Young MSM aged 15 to 24 years | “MyChoices”: a mobile app-based intervention  Core element included: information about PrEP and links to HIV prevention services at local clinical sites, links to videos related to PrEP (eg, what is PrEP and talking to your doctor about PrEP) | Social cognitive theory | Standard of care: Participants will receive written prevention material including recommendations for HIV testing and referrals to local HIV testing sites and prevention services. | Participants will be followed for 6 months, with follow-up assessments collected at baseline, 3 and 6 months | (1) PrEP uptake: proportion of those with a behavioral indication for PrEP who are prescribed and utilize PrEP and (2) PrEP use self-efficacy | Acceptability:  Mean System Usability Scale (0-100) score: 71±11.8  Feasibility:  Frequency of using the app: 8 times on average over the 2 months |
| Pilot trial: Biello et al. (2021) [64] |  |  |  |  |  |  |  |  |  |  |  |
| Blumenthal et al. (2019) [54] | San Diego, CA | April 2014 to June 2016 | RCT | Participants were recruited from three San Diego HIV testing sites. | MSM only  Age (median): 32  White: 60%  Latino: 29% | “PrEPARE2”:  The baseline survey generated a calculated HIV risk score, estimating HIV risk based on reported condomless anal intercourse and sexually transmitted infections, and was provided to individuals in the intervention arm. | Not reported | Participants were not given calculated HIV risk score. | Baseline: 86 vs. 85  Week 8: 76 vs. 59  Week 12: 61 vs. 58 | (1) Initiation of PrEP at week 8 | (1) Initiation of PrEP at week 8: 11% VS. 10%, p>0.99 |
| Liu et al. (2019) [70] | Not reported | Launched in October 2018 | RCT | Web-based and social media strategies (Craigslist, social networking ads, etc.); distributing posters, flyers, and palm cards; direct outreach at local venues frequented by MSM; and clinic-based recruitment including reviewing medical charts or referrals | MSM aged 15 to 24 years | “LYNX”: a mobile app-based intervention  The app included: PrEP educational materials, testimonials of peers who decided to take PrEP, impact of PrEP on Sex Pro score, links to youth clinics offering PrEP, app-based tips for insurance/access issues and PrEP navigation through app chat function | Information motivation behavior skills model | Standard of care:  Participants will be provided with a list of testing sites, along with an informational brochure about PrEP. | Participants will be followed for 24 weeks with follow-up assessments conducted at 12 and 24 weeks. | (1) PrEP knowledge, attitudes, motivations, and behavioral skills related to PrEP uptake; and (2) PrEP linkage measured by: the proportion of participants reporting interest in PrEP uptake during follow-up, the proportion making and attending a clinic appointment for PrEP evaluation, the proportion who receive a prescription for PrEP and the proportion who pick up PrEP medication from the pharmacy | The trial is ongoing. |
| Liu et al. (2019) [24] | Chicago, IL | April 2015 to March 2016 | RCT | Clinical-based recruitment; online ads; and provider referrals | Young MSM  Age (range): 18–29  Black: 27%  Latino: 36% | PrEPmate: a youth-tailored, bidirectional text-messaging  Intervention  The SMS-based adherence support component includes weekly “check in” messages, and daily pill-taking reminder messages sent at a customized time. Online components included a password-protected website providing access to key information about PrEP an online support forum. | Information motivation behavioral theory | Standard of care: a risk assessment, PrEP education, and brief adherence and risk-reduction counseling, clinical evaluation, medical management, and PrEP dispensation, and access to a pager to reach a clinician. | Baseline: 81 vs. 40  Week 4: 96% vs. 88%  Week 12: 86% vs. 75%  Week 24: 81% vs. 65%  Week 36: 80% vs. 57% | PrEP adherence: TFV-DP concentrations ≥700 fmol/punch (consistent with ≥4 doses/week) | Acceptability:  (1) PrEPmate to be very/somewhat helpful: 88%  (2) recommend PrEPmate to others: 92%  Efficacy:  Protective TFV-DP levels: 72% vs. 57% (OR=2.05, 95% CI: 1.06–3.94) |
| Siegler et al. (2019) [37] | Rural areas of Georgia, Mississippi, and North Carolina | Launched in May 2019 | RCT | Banner ads and brief electronic messages on geospatial networking apps and social media platforms; and peer referral | Young MSM aged 18 to 24 years | Home-care intervention: a mobile app-based intervention  Participants will receive a study app (eP) that incorporates a messaging platform, a scheduling and milestone-based tracking system for PrEP care progress, electronic behavioral surveys, and interactive video consultations with a clinician. | Andersen behavioral model adapted to HIV care | Standard of care:  Participants will receive a listing of nearest local PrEP providers to receive standard PrEP care. | Participants will be followed for 12 months, with follow-up assessments collected at 1, 3, 6, 9 and 12 months. | (1) Protective levels of the active metabolite of oral PrEP (TFV-DP) drug levels at the 12-month study visit; (2) initiation into PrEP care based on self-report and pill bottle photos; (3) maintenance in PrEP care at the study midpoint and (4) changes in PrEP indication | The trial is ongoing. |
| Starks et al. (2019) [46] | New York City, NY | Launched in February 2018 | RCT | Web-based recruitment | Young partnered MSM aged 18 to 29 years | “PARTNER”:  A 4-session intervention that integrates video-based communication training to address drug use and HIV prevention among partnered young MSM | Motivational interviewing and couples interdependence theory | Attention-matched psychoeducation control: Participants are provided with information about HIV-risk reduction, PrEP, and substance use. | Participants will be followed for 12 months, with follow-up assessments collected at 3, 6, 9 and 12 months. | (1) Self-reported PrEP uptake; and (2) PrEP adherence measured by a timeline follow-back interview approach and nail samples | The trial is ongoing. |
| Sullivan et al. (2019) [47] | Detroit, MI; New York City, NY; and Atlanta, GA | January -November 2018 | RCT | Targeted banner ads (eg, Facebook); traditional print ads (eg, flyers, public transit); recruitment at venues; referrals from community service providers; and in-person outreach | MSM aged 18 years and older | “M-Cubed”: a mobile app-based intervention  The intervention provided risk-customized written and video messages for participants. The messages included condom use, HIV/STI testing, PrEP, etc. | Social cognitive theory | Wait-list control:  Participants were given the option of accessing the intervention app at nine months postenrollment. | A total of 1229 MSM were enrolled. Participants were followed for 9 months with follow-up assessments conducted at 3, 6 and 9 months. | PrEP use and adherence | The result has not been reported. |
| Kuhns et al. (2020) [61] | Nationwide | Not reported | RCT | Local recruitment (Birmingham, AL, Chicago, IL, New York City, NY, Seattle, WA):  youth- and sexual minority-focused community organizations and events and posted flyers; and nationwide recruitment: ads on platforms frequented by adolescents | Racially and ethnically diverse young MSM aged 13 to 18 years | “MyPEEPs”: a mobile app-based intervention  MyPEEPs provides educational information about HIV and STIs, raises awareness about minority stress, and builds skills for condom use, emotion regulation, and negotiating interpersonal and substance related risk. The content is delivered through a series of games, scenarios and role-plays within 21 mobile activities. | Social-personal framework | Delayed intervention: Participants will be provided with access to the MyPEEPS Mobile app at the 9-month visit. Procedures for app access and incentives for completion are the same as for the intervention condition. Access is provided through the 12-month study visit. | Participants will be followed for 12 months with follow-up assessments conducted at 3, 6, 9 and 12 months (delayed intervention arm only). | Uptake of PrEP | The trial is ongoing. |
| Muessig et al. (2020) [62] | Nationwide | Launched in July 2020 | RCT | Ads based on sociodemographic characteristics on social media sites (e.g., Facebook, Tumblr, Instagram, Black Gay Chat Live, Jack’d, Grindr, and Scruff); clinic referrals; and participant repositories | Black and Latinx MSM aged 15 to 29 years | “HealthMpowerment 2.0” (HMP 2.0): an app- and peer- based intervention  (1) Researcher-created network intervention: Participants have access to all features of HMP 2.0: resource center, test kit ordering, care navigator, profile, activities, forums, etc.  (2) Peer-Referred Network Intervention: Participants have access to all features of HMP 2.0 and a customized invitation for peers to join the study. | Integrated behavior model | Information-only control:  Participants have access to informational content only: resource center, test kit ordering and care navigator | Participants will be followed for 12 months with follow-up assessments conducted at 3, 6, 9, and 12 months. | Uptake and maintenance of PrEP | The trial is ongoing. |
| Mustanski et al. (2020) [63] | Nationwide | Launched in April 2018 | RCT | Ads on social media (eg, Instagram, Facebook); active web-based engagement using geospatial dating apps; and other social media outlets (eg, Reddit, Tumblr) | MSM aged 13 to 18 years | “SMART”: a web-based stepped-care intervention  SMART Sex Ed (SSE, tier 1): an information-only intervention to which all participants will be granted access; SMART Squad (tier 2): a more intensive selective intervention offered to those who report HIV risk intentions or behaviors following SSE; SMART Sessions (tier 3): a higher cost indicated intervention designed for those who continue to report HIV risk intentions or behavior following the two previous interventions. | Medicine’s prevention model, information motivation behavior skills model and motivational interviewing | SSE 2.0: an expanded version of the SSE (with 6 modules and 2 boosters) | Participants were followed for 12 months with follow-up assessments conducted at 3, 6, 9, and 12 months. | PrEP knowledge, current and past 3-month PrEP use, PrEP adherence, motivation to start PrEP, and reasons for discontinuation | The trial is ongoing. |
| Rouffiac et al. (2020) [39] | Jackson, MS | Launched in February 2018 | RCT | Flyers at local STI/HIV testing clinics and word of mouth | Black MSM  Age(mean±SD): 23.9±4.8  High school graduate or GED: 62% | A mobile health intervention:  The intervention includes text messages with publicly available internet content (websites and YouTube videos) that provide factual information, motivational materials, and behavioral skills related to PrEP and HIV prevention. | Information motivation behavior skills model | Enhanced standard of care:  Patients are given a CDC informational handout with basic PrEP facts; shown a brief online video about PrEP; and given contact information for the clinic care coordinator. | 65 participants were randomized. Follow-up assessments will occur at enrollment and at 4 and 16 weeks. | (1) Attendance at PrEP services appointment; (2) receipt of PrEP prescription; (3) PrEP knowledge; (4) motivation for engaging in PrEP care: IMB PrEP Care Motivation Scale, Rollnick’s Readiness Ruler; and (5) self-efficacy for PrEP care | The trial is ongoing. |
| Protocol: Whiteley et al. (2020) [40] | Jackson, MS | 2017 to 2019 | RCT | Clinical-based recruitment: patients attending the PrEP clinics | Cis-gendered MSM  Age(mean±SD): 25.1±4.2  Black/African American: 85.5%  Homosexual: 68.1% | “Viral Combat”: an iPhone game-based intervention  During game play, participants fight off HIV and keep it from entering the body. Players engage with providers, take medication, and make healthy decisions to move to the next level. Tailored messages from doctors, clinicians, and friends are integrated as the player successfully destroys virus and collects pills. | Social learning theory (information, motivation, behavioral skills) | Participants received a non-PrEP related mobile game on a smart phone provided by the study. | Baseline: 81  Week 12: 63  Week 24: 55 | (1) Biological outcome of adherence: intracellular TFV–DP measured in red blood cells using DBS; and (2) behavioral outcomes: self-report of PrEP adherence; perception of the ability to perform the necessary PrEP skills; and social support for PrEP treatment | (1) Engagement in optimal PrEP dosing at 24 weeks (intervention vs. control): OR = 3.75 (95% CI: 1.20–11.77, p = 0.02)  (2) no significant between-group differences in other behavioral or psychosocial outcomes |
| Efficacy evaluation: Whiteley et al. (2021) [66] |  |  |  |  |  |  |  |  |  |  |  |
| Reback et al. (2021) [72] | A west coast metropolitan | Launched in January 2019 | RCT | Banner ads or digital flyers placed on gay websites, apps, and social media sites that target MSM; street- and venue-based outreach; poster ads; and participants referral | Methamphetamine-using MSM | “Getting Off”: a mobile app-based intervention  Core elements include treatment and recovery structure, meaning of meth use, triggers, social networks, emotions and feelings, sex and HIV, sexual identity, relapse prevention and behavioral assessment. participants will have immediate access to the Getting Off app. | Stages of change model and cognitive behavioral therapy model | Delayed delivery arm:  Participants will have access to the Getting Off app after a delayed 30-day period | Participants will be followed for 9 months with follow-up assessments conducted at 1, 2(delayed delivery arm only), 3, 6 and 9 months. | (1) PrEP uptake (dichotomous) and (2) DBS analysis for intraerythrocytic tenofovir-diphosphate | The trial is ongoing. |
| da Silva et al. (2021) [52] | Chicago, IL | November 2015 to November 2017 | RCT | Network services (partners services and social network strategy services) | Black MSM  Age (median): 25.9 (IQR: 22.5-28.5)  Gay: 62% | Partner Services PrEP: a 60-minute face-to-face session with an interventionist + 4 minibooster sessions via phone or text message  Core components included: interactive education on HIV and PrEP; assessment of participant’s intrinsic motivation to reduce HIV risk and engage in PrEP care; a personalized plan to navigate linkage to PrEP care; and identification of significant barriers in the plan. | Information motivation behavior skills model and motivational interviewing techniques | Control participants received treatment as usual: a brief risk assessment, referral for additional STI testing, and provision of HIV prevention programing, including PrEP care. | Baseline: 74 vs. 70  Month 3: 57 vs. 51  Month 12: 50 vs. 47 | (1) Linkage to PrEP care: having at least one outpatient clinic visit within 3 months of the baseline session (eg, linkage window) where PrEP was discussed with a PrEP prescriber regardless of whether PrEP was initiated; (2) PrEP initiation within the linkage window and within the study period; (3) time to linkage to PrEP care; and (4) time to PrEP initiation | (1) Linked to PrEP care within linkage window: 24% vs. 11%, P = 0.04  (2) PrEP initiation within the linkage window: 24% vs. 11%, P=0.05  (3) Days to PrEP care linkage: 26.5 (6.0–141.8) vs. 191.5 (21.5–297.0), P =0.05  (4) Days to PrEP initiation: 61.0 (6.0–253.0) vs. 209.0 (20.0–327.0), p=0.30 |
| Weitzman et al. (2021) [49] | Greater Boston area, MA | Not reported | Pretest-posttest | Ads posted on Facebook, Craigslist, UserInterviews, and two medical centers located in Boston | Young MSM  Age (mean±SD): 25.6±2.27  Caucasian: 52%  African American: 19%  Asian: 19% | “Dot”: a mobile app-based intervention  The Dot intervention combined personalized pill reminders with positive psychology-based texts designed to both encourage PrEP adherence, and provide PrEP education to culturally-diverse Young MSM. | Not reported | Pretest assessment of the intervention for PrEP adherence | Baseline: 54  Week 6: 54 | (1) A 3-item self-reported PrEP adherence (2) PrEP treatment self-efficacy: how confident participant felt about carrying out PrEP treatment-related behaviors in the last month; and (3) PrEP knowledge: medication purpose, side effects, concomitant condom use and HIV testing | Pretest vs. posttest  (1) Perfect adherence rate (mean): 0.39 vs. 0.72, p < 0.001  (2) PrEP treatment self-efficacy (mean): 65.92 vs. 70.65, p = 0.004  (3) PrEP knowledge (mean): 6.88 vs. 6.91, p = 0.784 |

Table S2. Summary of study characteristics: interpersonal-level interventions

| Source | Location/setting | Recruitment/Study period | Study design | Recruitment strategy | Population characteristics | Intervention (components) | Theoretical/conceptual framework | Control | Sample size & retention (intervention vs. control) | Outcome measures | Findings (acceptability, feasibility, efficacy or cost-effectiveness) |
| --- | --- | --- | --- | --- | --- | --- | --- | --- | --- | --- | --- |
| Patel et al. (2018) [27] | New York, NY | Launched in June 2017 | Cluster RCT | Peer leaders were recruited through ads via emails to local youth and LGBTQ-focused community organizations, word-of-mouth through key informants in local young MSM communities, and targeted ads on Facebook and Instagram. Peer leaders recruited network study participants via their existing online social networks. | Young Black and Latinx Gay, bisexual, and other MSM aged 18 to 29 years | “Empowering with PrEP (E-PrEP)”: a social media–based social network intervention  Peer leaders launched the intervention by disseminating online messaging on Facebook or Instagram to provide PrEP education to their existing online networks. | Diffusion of innovation and information motivation behavior skills model | Attention-matched general health control (E-Health):  E-Health focused on a broad range of health topics prioritized by the peer leaders, but did not include any contents about HIV or PrEP. | Participants will be followed for 12 weeks, with follow-up assessments collected at 6 and 12 weeks. | (1) self-reported PrEP uptake or intention (measured by indicating either current use of PrEP or intention to use PrEP in the next month); (2) change on the PrEP motivational cascade at 12 weeks; (3) PrEP knowledge; PrEP-related stigma; and attitudes about PrEP. | The trial is ongoing. |
| Protocol: Young et al. (2018) [25] | Chicago, IL | 2016 to 2018 | RCT and crossover | Respondent-driven sampling draws on peer referrals | Young Black MSM  Age (range): 18–35  Gay: 61% | “PrEP Chicago”: a social network intervention  The intervention is designed to develop a participant’s knowledge about PrEP and their willingness to discuss it with others. The intervention includes: (1) training workshop (HIV tutorial, specifics of PrEP and communication skills to increase the effectiveness as peer change agents); and (2) booster session.  Participants were randomly assigned to: (1) intervention in Year 1 + attention control in Year 2 or (2) attention control in Year 1 + treatment in Year 2. | Not reported | The attention control consists of a sex diary activity designed to help participants assess sexual risk. | Baseline: 209 vs. 214  Week 55: 177 vs. 165  Week 110: not reported | (1) The number of individuals successfully referred to a PrEP information line; (2) the number of individuals who makes an initial clinic appointment; (3) PrEP knowledge and attitudes; (4) perceived PrEP barriers; and (5) intended and actual PrEP uptake | Interim analyses before crossover assignment:  PrEP referral was most likely to occur within 3 days of an intervention session compared to control (OR = 0.07, 95% CI: 0.02–0.013) |
| Interim analyses: Schneider et al. (2021) [68] |  |  |  |  |  |  |  |  |  |  |  |
| Desrosiers et al. (2019) [34] | Washington, DC | August 2016 to February 2017 | RCT | Social media-based recruitment: Jack’d, Grindr, Tinder and Adam4Adam | Black young MSM  Age (range): 16–25 | A culturally-tailored counseling intervention:  Participants received personalized comprehensive counseling from a staff member who self-identified as a Black MSM. The session involved PrEP education, sexual risk reduction counseling, assessing and addressing perceived barriers to initiating PrEP. | Client-centered care  coordination approach based on self-determination theory of human motivation | Participants were seen by a health care provider who provided the participants PrEP education. | Baseline: 25 vs. 25  Month 3: 25 vs. 23 | (1) Has seen a medical provider in the last 3 months; (2) has taken PrEP in the last 3 months; (3) currently taking PrEP; (4) has talked to a medical provider about PrEP in the last 3 months; (5)  plans to continue taking PrEP; (6) plans to begin taking PrEP within the next 3 months | (1) Has talked to a medical provider about PrEP in the last 3 months: 85% vs. 42%, p = 0.041  (2) Has taken PrEP in the last 3 months: 24% vs. 0%, p = 0.023  Other outcomes were nonsignificant. |
| Gamarel et al. (2019) [60] | Nationwide | Launched in June 2017 | RCT | Online ads placed on key social media websites (eg, Facebook) and social media sites aimed specifically at MSM (eg, Grindr) | MSM aged 15 to 19 years and their partners | “We Prevent”: a couple- and video chat-based intervention  Two telehealth-delivered sessions are: (1) techniques to explore and build communication skills in a relationship; and (2) couples HIV testing and counseling and prevention planning. Both sessions are attended by both members of the dyad. | Relationship-oriented information motivation behavioral skills model and motivational interviewing techniques | Participants will engage in only 1 telehealth session: the existing couples HIV testing and counseling intervention delivered via video counseling. | Participants will be followed for 9 months, with follow-up assessments conducted every 3 months. | (1) PrEP awareness: whether the participant has heard of PrEP; (2) PrEP willingness: likelihood of PrEP use across different conditions (eg, partner types and experiencing potential side effects); (3) PrEP uptake: whether they have begun using PrEP; and (4) self-reported adherence to PrEP. | The trial is ongoing. |
| Arrington-Sanders et al. (2020) [38] | Baltimore, MD; Philadelphia, PA; and Washington, DC | Launched in September 2017 | RCT | Flyers directly given to youth from study staff at a clinical site or at a community-based organization or in-person events; flyers posted at clinical sites that serve LGBTQ youth or at the offices of community-based organizations; and ads placed on webpages and social and sexual networking sites frequented by MSM | Young Black and Latinx MSM and Young Black and Latinx transgender women aged 15 to 24 years | A peer-based mobile-enhanced intervention:  Participants will meet with their coach every 3 months and have weekly telephonic check-ins, to review progress on personal goals, risk reduction, and help to troubleshoot factors that may increase HIV risk or personal goals. Coaching is tailored to the participants’ specific needs and case management or navigation needed. | Motivational interviewing | Standard of care:  Participants are provided information about HIV prevention strategies and the schedule for follow-up data assessments. | Participants will be followed for 12 months, with follow-up assessments collected at baseline, 3, 6, 9, and 12 months, and an exit interview at 18 months. | (1) Self-reported PrEP use over 12 months in PrEP users | The trial is ongoing. |
| Colson et al. (2020) [48] | New York, NY | March 2015 to March 2017 | RCT | Referrals from other studies; current HIV-negative Harlem United clients; targeted direct recruitment strategies, including ads/notices placed on Facebook, dating websites for Black gay men, and Craigslist; posters and flyers at LGBT and HIV organizations and at NYC STI clinics; street outreach; and public events such as Gay Pride parades | Black MSM and TGW  Age 18-29: 44.6%  Age 30-49: 39.2%  Age 50+: 16.2%  Latinx: 20.6%  Homosexual: 48.5%  Bisexual: 48.5% | Enhanced PrEP adherence support: a tailored mobile health intervention  Components: (1) peer navigation (providing information and social support), (2) a PrEP-focused in-person support group, (3) an online support group using social media, and (4) automated text message reminders. Participants could select which components they used, and at what intensity. | Not reported | Standard of care | Baseline: 101 vs. 103  Month 3: 67 vs. 64  Month 6: 67 vs. 61  Month 9: 63 vs. 62  Month 12: 69 vs. 63 | Self-reported once-daily oral PrEP adherence measured by: (1) how many days in the last 30 days participants missed taking PrEP, (2) how well they did in the last 3 months taking PrEP the way they were supposed to, and (3) how often they took PrEP in the way they were supposed to in the last 3 months. | No statistically significant differences between arms were noted at any timepoint. |
| Edwards et al. (2020) [55] | Los Angeles County Men’s Central Jail and residential recovery facilities in the county | Launched in November 2019 | RCT | Referral by staff at residential facilities and in-person enrollment in jail | MSM and transgender women leaving jail | A mobile app- and peer-based intervention:  Participants will receive customized wellness goals in addition to GeoPass (a GPS-based mobile app), cash incentives, and the support of a trained peer mentor for 6 months. | Social cognitive theory and motivational interviewing | Participants receive usual care. | Participants will be followed for 9 months with follow-up assessments conducted at 3, 6 and 9 months. | Being prescribed and adhering to PrEP for HIV | The trial is ongoing. |
| Harawa et al. (2020) [56] | Los Angeles, CA | October 2015 to April 2017 | RCT | Direct outreach at public venues, community-based organizations, parks, and events; provider referrals; fliers placed at public venues; and online recruitment via Craigslist.com, Instagram, and a study website | Black MSM  Age(mean±SD): 44.3± 11.2  Completed high school: 72% | “The Passport to Wellness”: a peer-based intervention  Intervention components included: (1) a customized wellness plan (or Passport) that included referrals to health and support services and incentives for accessing those services; (2) incentives for providing documentation of completed Passport activities; (3) a trained peer who provided support, encouragement, and navigation; and (4) social/education group outings. | Motivational interviewing, principles of patient navigation and contingency management, social impact, social comparison, and social cognitive theories | Non-peer mentor intervention arm | Baseline: 55 vs. 50  Month 1: 23 vs. 18  Month 4: 23 vs. 18  Month 6: 34 vs. 27 | Awareness and use of PrEP | (1) Has ever heard of PrEP at 6 months: 34% vs. 24%  (2) Has taken part in an informational session about PrEP at 6 months: 31% vs. 19% |
| Kelly et al. (2020) [26] | Milwaukee, WI | 2016 to 2017 | Pretest-posttest | Seeds were identified by community outreach (clubs, hangout places, and drop-in centers for racial minority LBGT youth). Seeds were asked to give MSM friends study invitation packets. | Black MSM  Age (mean): 27 African American/Black: 89%  High school education or less: 73% | A social network-level intervention  Persons most socially interconnected with others in each network were identified as leaders, and they attended a 7-session intervention that provided training to increase knowledge about PrEP and its benefits, address misconceptions and concerns about PrEP, endorse PrEP use as a symbol of pride and health, and deliver these messages to others in their social networks between sessions. | Reasoned action and social cognitive theory | PrEP outcomes at baseline | Baseline: 37  Month 3: 33 | (1) PrEP knowledge;  (2) PrEP attitudes; (3) PrEP stigma; (4) PrEP descriptive norms; (5) PrEP subjective norms; (6) PrEP self-efficacy; (7) PrEP behavioral intentions; and (8) willingness to use PrEP | Acceptability:  Willing to attend sessions about PrEP: 97%  Efficacy (baseline vs. month 3):  (1) knowledge: 0.48 vs. 0.73  (2) attitudes: 4.04 vs. 4.41  (3) stigma: 1.81 vs. 1.76  (4) descriptive norms: 3.88 vs. 4.14  (5) subjective norms: 4.19 vs. 4.59  (6) self-efficacy: 3.59 vs. 3.71  (7) intentions: 3.22 vs. 3.43  (8) willingness: 4.40 vs. 4.78 |
| Blashill et al. (2021) [58] | San Diego County, CA | Launched in August 2019 | RCT | FHCSD outreach and HIV/STI testing programmes; gay- friendly events; flyering local LGBT community centres, gay- identified/ friendly coffee shops, gyms and bars; targeted ads through Facebook and Instagram; and the use of geolocation social networking mobile applications tailored to sexual minority men (eg, Grindr, Scruff and Jack’d). | Latino MSM aged 18 and 29 years | Patient navigation intervention: a peer-based intervention  Two part- time Spanish–English bilingual peer lay navigators will provide the intervention during the study. The intervention includes an introductory module, five educational modules (HIV prevention, PrEP introduction, PrEP efficacy, PrEP side effects and PrEP adherence) and a module focused on decision support. | Not reported | Usual care intervention:  Immediately following Participants will be provided CDC’s two- page PrEP Information Sheet in the participant’s preferred language (either English or Spanish). | Participants were followed for 6 months with follow-up assessments conducted at 3 and 6 months. | Seven behaviors associated with the PrEP care continuum (i.e., appointment scheduled for PrEP consultation; appointment attended for PrEP consultation; PrEP prescription received; PrEP prescription filled; PrEP initiated; self- reported PrEP adherence over the past 7 days; and PrEP follow- up medical appointment attended) | The trial is ongoing. |

Table S3. Summary of study characteristics: community-level interventions

| Source | Location/setting | Recruitment/Study period | Study design | Recruitment strategy | Population characteristics | Intervention (components) | Theoretical/conceptual framework | Control | Sample size & retention (intervention vs. control) | Outcome measures | Findings (acceptability, feasibility, efficacy or cost-effectiveness) |
| --- | --- | --- | --- | --- | --- | --- | --- | --- | --- | --- | --- |
| Doblecki-Lewis et al. (2019) [35] | South Florida | January 2016, to October 20 2016 | RCT | Study flyers distributed inside outpatient clinics; and message broadcasted locally through GRINDR | MSM/transgender women (54%) and heterosexual men and women  Hispanic/Latino: 43%  Black/African American: 34%  Less than a high school education: 44% | Active strengths-based case management intervention: a personalized intervention  Activities during the first visit with the patient navigator included building the relationship; emphasizing personal strengths; learning to make contact. Participants had 4 optional follow-up visits to revisit personal strengths, reevaluate available resources, and focus on the remaining elements of reviewing progress and completing the work. | Not reported | Passive referral:  Participants were provided with a package of information including PrEP providers in the area, recommendations regarding initiating PrEP discussion with the provider and information on patient assistance and co-pay assistance programs for PrEP. | Baseline: 30 vs. 31  Loss to follow up at week 6 or 12: 18 vs. 15 | (1) Completion of provider visit; (2) initiation of PrEP; and (3) time to initiation of PrEP | (1) Completion of provider visit: 53.3% vs. 32.2%, p = 096  (2) initiation of PrEP: 40% vs. 29%, p = 0.367  (3) mean time to initiation of PrEP: 12.76 vs. 13.42 weeks, P = 0.382 |

Table S4. Summary of study characteristics: healthcare system-level interventions

| Source | Location/setting | Recruitment/Study period | Study design | Recruitment strategy | Population characteristics | Intervention (components) | Theoretical/conceptual framework | Control | Sample size & retention (intervention vs. control) | Outcome measures | Findings (acceptability, feasibility, efficacy or cost-effectiveness) |
| --- | --- | --- | --- | --- | --- | --- | --- | --- | --- | --- | --- |
| Mayer et al. (2017) [41] | Boston, MA | November, 2012 to December 2013 | RCT | Community outreach by the staff of Fenway Health, a Boston community health center; ads on social media; and flyers posted within the waiting areas of Fenway Health’s clinical care sites | MSM only  Age(mean±SD): 38.24±12.6  White: 86%  Employed full-time: 70% | “Life-Steps for PrEP”: a cognitive behavioral intervention  The intervention entailed four nurse-delivered initial and two booster sessions based on Life-Steps, an ART treatment adherence intervention. | The principles of cognitive-behavioral therapy, problem-solving therapy and motivational interviewing | Time and session-matched comparison counseling intervention provided information and supportive counseling. | Baseline: 25 vs. 25  Month 3: 21 vs. 20  Month 6: 19 vs. 20 | (1) PrEP adherence: measured by Wisepill, an electronic pill storage device that allows for realtime adherence monitoring; and (2) Tenofovir plasma levels collected at the 3- and 6-month follow-up visits | (1) Wisepill adherence was high in both groups, and not statistically different.  (2) Plasma tenofovir levels were significantly higher in the intervention group at 6 months (p = 0.037). |
| Raifman et al. (2018) [43] | Rhode Island STD Clinic | January 1, 2012 to December 31, 2015 | Quasi-experimental | Participants were recruited from patients attending the clinic. | MSM only  Age 20–24: 27%  Age 25–29: 19%  Age 30–34: 17%  Age 35–44: 11%  Non-Hispanic white: 64%  Hispanic/Latino: 16% | Clinical PrEP education intervention:  In a private clinic room in a one-to-one counseling session, clinic staff provided patients with information on what PrEP is, who should consider it, and commonly asked questions. They also referred patients who were interested in PrEP care. | Not reported | HIV-negative men who have sex with women only who did not receive the PrEP education intervention | Baseline: 234 vs. 82 | (1) Self-reported PrEP awareness: have you heard of taking HIV medications to prevent infection in people who are HIV negative? (yes/no); and (2) PrEP use: have you ever taken pre-exposure prophylaxis (yes/no) | (1) Change in PrEP awareness: 27.2 vs. 13.7 percentage points  (2) change in PrEP use: 7.1 vs. 2.4 percentage points |
| Parsons et al. (2019) [36] | Miami, FL; Detroit, MI; and Philadelphia, PA | Launched in November 2018 | RCT | Three Subject Recruitment Venues (Wayne State University, University of Miami, and Children’s Hospital of Philadelphia) | Young MSM aged 15 to 24 years | Delivery of the Young Men’s Health Project (YMHP) intervention in person by community health workers at the clinic site:  YMHP sessions included: building motivation for behavior change, setting goals on behaviors and discussion about how PrEP fits within a youth’s goal for healthy behaviors. Both intervention and control groups involve completion of the 4 YMHP sessions and the delivery of PrEP information and navigation services to interested participants. | Motivational interviewing | Delivery of intervention by phone or video chat using apps such as Skype or FaceTime. | Participants will be followed for 12 months, with follow-up assessments collected at baseline, immediate postintervention, 3, 6, 9 and 12 months. | Motivational PrEP Cascade: a series of 21 questions to assess PrEP treatment uptake and adherence (familiarity with PrEP; experiences and acceptability; and PrEP contemplation, preparation,  action, and maintenance) | The trial is ongoing. |
| Chan et al. (2021) [69] | Not reported | January 2019 to March 2020 | RCT | Clinic-based recruitment | MSM only  Age (mean±SD): 32.0± 13.0  Non-Latinx White: 66.7%  Latinx: 26.8%  Same-gender loving or gay:79.1% | A brief behavioral intervention:  Participants were offered a brief (15–20 minutes) intervention delivered by state-certified STI clinic counselors at the time of STI testing to address barriers to PrEP uptake. The initial session was followed by a brief, telephone booster session that lasted <10 minutes. | Motivational interviewing | Participants received treatment-as-usual services that included referral to PrEP services. | Baseline: 44 vs. 43  Month 1: 33 vs. 34  Month 3: 33 vs. 32  Month 6: 26 vs. 26 | (1) Scheduling an appointment with a clinic prescriber to discuss PrEP, (2) attending the prescriber appointment, and (3) accepting a PrEP prescription | Intervention vs. control (OR and 95% CI)  (1) Schedule an appointment: 6.0 (2.3-15.6)  (2) Attend the prescriber appointment: 3.6 (1.5-8.9)  (3) Receive and accept a prescription for PrEP: 3.6 (1.5-8.9) |

Table S5. Summary of study characteristics: multilevel interventions

| Source | Location/setting | Recruitment/Study period | Study design | Recruitment strategy | Population characteristics | Intervention (components) | Theoretical/conceptual framework | Control | Sample size & retention (intervention vs. control) | Outcome measures | Findings (acceptability, feasibility, efficacy or cost-effectiveness) |
| --- | --- | --- | --- | --- | --- | --- | --- | --- | --- | --- | --- |
| Bauermeister et al. (2018) [31] | Philadelphia, PA; Atlanta, GA; and Houston, TX | Launched in September 2016 | RCT | Broad range of social media outlets | Young MSM aged 15 to 24 years | “Get Connected 2.0”: a mobile-optimized web app-based intervention  Participants will be granted access to a Web app with content tailored to their specific demographic characteristics, HIV and STI risk behaviors, and sociocultural context. | Self-determination theory, integrated behavioral model and motivational interviewing principles | Participants will be directed to the AIDSVu.org testing site locator. | Participants will be followed for 12 months with follow-up assessments conducted at 1, 3, 6, 9 and 12 months. | (1) PrEP awareness: a single-item measure of whether the participant has heard of PrEP; (2) PrEP willingness: how likely the participant would be to start PrEP in the next 3 months and the reason(s) why the participant is not currently taking PrEP; (3) PrEP uptake: whether the participant has begun using PrEP; and (4) adherence to PrEP | The trial is ongoing. |
| Arnold et al. (2021) [57] | San Francisco Bay Area, CA | September 2018 to September 2019 | Pretest-posttest | In-person recruitment at balls; street-based outreach at gay pride and other LGBT community events; social networks and peer referrals; and social media (such as Facebook and Instagram) | MSM (55%) and transgender individuals  Age (range): 19–67  Black/African-American:61%  Latinx: 9% | “We Are Family”:  Four components included in-person group session about HIV education; community-level events; mhealth tool (app); and dedicated service provider who provided HIV related services. | Not reported | PrEP outcomes at baseline | Baseline: 118  Month 6: 114 | (1) Currently taking PrEP; (2) PrEP-adherent past 6 months; and (3) extremely likely to use PrEP in next 6 months: 59.0% vs. 55.4% | (1) Willing to refer a friend to the app: 86%  (2) Found the app to be personally relevant: 65%  (3) Baseline to follow-up change  Currently taking PrEP: 27.7% vs. 24.3%  PrEP-adherent past 6 months: 9.8% vs. 12.2%  Extremely likely to use PrEP in next 6 months: 59.0% vs. 55.4% |
